# Oncostatin M reduces atherosclerosis development in APOE*3Leiden.CETP mice and is associated with increased survival probability in humans

**DOI:** 10.1371/journal.pone.0221477

**Published:** 2019-08-28

**Authors:** Danielle van Keulen, Marianne G. Pouwer, Valur Emilsson, Ljubica Perisic Matic, Elsbet J. Pieterman, Ulf Hedin, Vilmundur Gudnason, Lori L. Jennings, Kim Holmstrøm, Boye Schnack Nielsen, Gerard Pasterkamp, Jan H. N. Lindeman, Alain J. van Gool, Maarten D. Sollewijn Gelpke, Hans M. G. Princen, Dennie Tempel

**Affiliations:** 1 Laboratory of Experimental Cardiology, University Medical Center Utrecht, Utrecht, The Netherlands; 2 Laboratory of Clinical Chemistry and Haematology, University Medical Center Utrecht, Utrecht, The Netherlands; 3 Quorics B.V., Rotterdam, The Netherlands; 4 TNO-Metabolic Health Research, Gaubius Laboratory, Leiden, The Netherlands; 5 Department of Cardiology, Leiden University Medical Center, Leiden, The Netherlands; 6 Icelandic Heart Association, Kopavogur, Iceland; 7 Faculty of Pharmaceutical Sciences, University of Iceland, Reykjavik, Iceland; 8 Department of Molecular Medicine and Surgery, Karolinska Institutet, Solna, Sweden; 9 Faculty of Medicine, University of Iceland, Reykjavik, Iceland; 10 Novartis Institutes for Biomedical Research, Cambridge, Massachusetts, United States; 11 Bioneer A/S, Hørsholm, Denmark; 12 Department of Vascular Surgery, Leiden University Medical Center, Leiden, The Netherlands; 13 TNO- Microbiology & Systems Biology, Zeist, The Netherlands; 14 Molecular Profiling Consulting, London, United Kingdom; 15 SkylineDx B.V., Rotterdam, The Netherlands; Universita degli Studi di Padova, ITALY

## Abstract

**Objective:**

Previous studies indicate a role for Oncostatin M (OSM) in atherosclerosis and other chronic inflammatory diseases for which inhibitory antibodies are in development. However, to date no intervention studies with OSM have been performed, and its relation to coronary heart disease (CHD) has not been studied.

**Approach and results:**

Gene expression analysis on human normal arteries (n = 10) and late stage/advanced carotid atherosclerotic arteries (n = 127) and *in situ* hybridization on early human plaques (n = 9) showed that *OSM*, and its receptors, OSM receptor (*OSMR*) and Leukemia Inhibitory Factor Receptor (*LIFR*) are expressed in normal arteries and atherosclerotic plaques. Chronic OSM administration in APOE*3Leiden.CETP mice (n = 15/group) increased plasma E-selectin levels and monocyte adhesion to the activated endothelium independently of cholesterol but reduced the amount of inflammatory Ly-6C^High^ monocytes and atherosclerotic lesion size and severity. Using aptamer-based proteomics profiling assays high circulating OSM levels were shown to correlate with post incident CHD survival probability in the AGES‐Reykjavik study (n = 5457).

**Conclusions:**

Chronic OSM administration in APOE*3Leiden.CETP mice reduced atherosclerosis development. In line, higher serum OSM levels were correlated with improved post incident CHD survival probability in patients, suggesting a protective cardiovascular effect.

## Introduction

Cytokines have an indisputable role in all stages of atherosclerosis development. In the initial stages of the disease, cytokines induce endothelial activation leading to endothelial adhesion molecule expression and leukocyte recruitment to the activated endothelium. In later stages of the disease, cytokines are involved in smooth muscle cell (SMC) migration, foam cell formation and enhanced MMP activity leading to plaque destabilization[1,2].

Similarly, a role for Oncostatin M (OSM) in atherosclerosis has been suggested[3,4]. This cytokine is secreted by activated macrophages and neutrophils and signals through the Leukemia Inhibitory Factor Receptor (LIFR) and the OSM receptor (OSMR)[5–7]. OSM induces endothelial activation by increasing cytokine release, adhesion molecule expression, and leukocyte adhesion to the activated endothelium in cultured endothelial cells[8–10]. Moreover, OSM reduces vascular integrity of rat blood brain barrier endothelial cells and enhances angiogenesis[11,12]. Next to its effects on the endothelium, OSM enhances SMC proliferation, migration and differentiation[4,12,13].

Additional evidence for this potential role of OSM in atherosclerosis, was provided by Albasanz-Puig *et al*., who showed that OSM is expressed in both murine and human atherosclerotic plaques[13]. Furthermore, in ApoE^-/-^ mice, OSMR deficiency attenuated atherosclerosis development and increased plaque stability[14].

Using a different approach, we recently demonstrated that short-term OSM administration (for 3 weeks) to APOE*3Leiden.CETP mice increased plasma E-selectin levels, Interleukin (IL)-6 mRNA expression in the aorta and Intercellular Adhesion Molecule 1 (ICAM-1) expression and monocyte adherence to the activated endothelium in the aortic root[10]. Collectively, these findings suggest that OSM may be involved in atherosclerosis development but so far this has never been studied.

The aim of this study is to investigate whether OSM is involved in atherosclerosis development in a humanized mouse model and in man. Therefore, we first investigated if OSM and its receptors are expressed in human normal and atherosclerotic arteries and if circulating OSM levels correlate with markers of endothelial activation in humans. Next, we explored the effect of long-term OSM administration on endothelial activation, atherosclerosis development and lesion composition in APOE*3Leiden.CETP mice, a translational model for human lipoprotein metabolism and atherosclerosis development[15]. Finally, we investigated if circulating OSM levels were associated with survival probability post coronary heart disease (CHD) in humans.

## Material and methods

### Microarray on BiKE study material

Late stage/Advanced atherosclerotic plaques were obtained from patients undergoing surgery for high grade (>50%) carotid stenosis and retained within the BiKE study. Normal artery controls were obtained from nine macroscopically disease-free iliac arteries and one aorta from organ donors without history of cardiovascular disease. All samples were collected with informed consent from patients or organ donor guardians. 127 plaques from BiKE patients and 10 normal arteries were analyzed by Affymetrix HGU133 plus 2.0 GeneChip microarrays. Robust multiarray average normalization was performed and processed gene expression data was transformed in log2-scale. The microarray dataset is available from Gene Expression Omnibus (GSE21545). The BiKE study cohort demographics, details of sample collection, processing, and analyses were previously described[16].

### *In situ* hybridization (ISH) on SOCRATES study material

Early stage atherosclerotic lesions for *in situ* hybridization were obtained from the SOCRATES biobank (Leiden University Medical Center, the Netherlands). Details of this biobank have been described previously[17]. Briefly, this biobank contains aortic wall patches obtained during kidney transplantation with grafts derived from cadaveric donors. Sample collection and handling were performed in accordance with the guidelines of the Medical and Ethical Committee in Leiden, the Netherlands, and the code of conduct of the Dutch Federation of Biomedical Scientific Societies (https://www.federa.org/?s=1&m=82&p=0&v=4#827). Chromogenic mRNA-ISH was essentially performed as previously described[18,19] on 9 atherosclerotic lesions from the SOCRATES biobank. For detection of the *OSM*, *OSMR* and *LIFR* mRNAs, ISH was performed in a Ventana Discovery ULTRA instrument (Ventana Medical Systems Inc., AZ, USA) using the ACD RNAscope^®^ 2.5 Red Kit (Advanced Cell Diagnostics, Newark, CA, USA) and the mRNA Discovery ULTRA RED 4.0 procedure. RNAscope^®^ 2.5 VS. Probes for Hs-OSM (#456389), Hs-OSMR-tv1 (#445699) and Hs-LIFR (#441029) were designed by the probe manufacturer (Advanced Cell Diagnostics). FFPE sections (5 μm) were applied to Superfrost Plus (Thermo Fisher Scientific) slides, and all operations including deparaffinization, pretreatment, ISH and counterstaining using hematoxylin were performed in a Ventana Discovery ULTRA instrument. Following the ISH-procedure in the Ventana instrument, slides were washed in lukewarm tap water with detergent until oil from the slides was fully removed. Subsequently, slides were washed in demineralized water, air dried and mounted in EcoMount mounting medium (Advanced Cell Diagnostics) prior to scanning in a bright-field whole-slide scanner (Axio Scan.Z1, Zeiss, Oberkochen Germany) using a 20x objective. The resulting digital images were inspected and regions of interest were selected.

### Proteomics on AGES-Reykjavik study material

Association between OSM levels and IL-6, vascular cell adhesion molecule (VCAM)-1, P-selectin, E-selectin, ICAM-1 and Monocyte chemoattractant protein-1 (MCP-1) levels, and between OSM levels and survival were explored in the AGES-Reykjavik cohort (n = 5457)[20], a single-center prospective population-based study of deeply phenotyped elderly European Caucasians (aged 66 through 96, mean age 75±6 years) who survived the 50-year-long prospective Reykjavik study. Phenotype description, patient numbers and other details related to the present study have been described previously[21]. The AGES-Reykjavik study was approved by the NBC in Iceland (approval number VSN-00-063), the National Institute on Aging Intramural Institutional Review Board (USA), and the Data Protection Authority in Iceland. We applied a custom version of the Slow Off-rate Modified Aptamer (SOMAmer) platform targeting proteins known or predicted to be found in the extracellular milieu, including the predicted extracellular domains of single- and certain multi-pass transmembrane proteins, as previously described[21].

For survival analysis post CHD, we used 698 incident CHD cases exhibiting 307 deaths during the survival follow-up period of 12 years. Follow-up time for survival post incident CHD was defined as the time from 28 days after an incident CHD event until death from any cause or end of follow-up time.

### Animals and treatments

Sixty-five female in-house bred APOE*3Leiden.CETP transgenic mice (10–15 weeks of age) were used. Mice were housed under standard conditions with a 12h light-dark cycle and free access to food and water. Body weight, food intake and clinical signs of behavior were monitored regularly. Mice received a Western type diet (semi-synthetic containing 15 w/w% cacao butter and 0.15% dietary cholesterol, Altromin, Tiel, the Netherlands). At t = 0 weeks, after a run-in period of 3 weeks, mice were matched based on body weight, age, plasma total cholesterol and E-selectin levels in 4 groups: a control group, and three intervention groups, two of which were treated with 10 or 30 μg/kg/day OSM for 16 weeks, and an initial priming group, which received 30 μg/kg/day OSM for the first 5.5 weeks only. All groups consisted of 15 mice except for the control group which had an additional 5 mice to monitor the atherosclerosis development. Five mice were removed from the study based on human end-point criteria and were excluded from all analyses: 2 mice in the 16 week 30 μg/kg/day OSM group and 1 in each of the other 3 groups. At t = 0 weeks, an ALZET^®^ Osmotic Pump Type 1004 (Durect, Cupertino, CA) containing either 10 or 30 μg/kg/day murine OSM (R&D systems, Minneapolis, MN) or the vehicle (PBS + 1% mouse serum) was placed subcutaneously in the flank and were replaced at t = 5.5 and 11 weeks. Doses were based on our previous research[10]. Prior to surgery, mice received the analgesic Carprofen (5 mg/kg s.c.) and were anesthetized with isoflurane (induction 4%, maintenance 2%). EDTA blood samples were drawn after a 4 hour fast at t = 0, 4, 8, 12 and 16 weeks for determination of total cholesterol and inflammatory markers. At t = 12 weeks, 4 mice from the control group were euthanized to assess atherosclerosis development for the determination of the end-point of the study. At t = 16 weeks, mice were euthanized by gradual CO_2_ inhalation. Death was confirmed by exsanguination (via heart puncture) and hearts were isolated. All animal experiments were performed conform the guidelines from Directive 2010/63/EU of the European Parliament on the protection of animals used for scientific purposes or the NIH guidelines. Approval was granted by the ethics committee on animal experiments (approval reference number DEC-3683) and the institutional animal welfare body (approval reference number TNO-255).

### Plasma parameters

Plasma cholesterol was measured spectrophotometrically with enzymatic assays (Roche Diagnostics). E-selectin and Monocyte Chemoattractant Protein 1 (MCP-1) were measured with ELISA kits from R&D (Minneapolis, MA, USA), and Serum Amyloid A (SAA) with an ELISA kit from Tridelta Development Limited (Maynooth, County Kildare, Ireland). All assays were performed according to the manufacturer’s instructions.

### Histological assessment of atherosclerosis and plaque composition

Atherosclerotic lesion area and severity were assessed in the aortic root area, as reported previously[22,23]. Briefly, the aortic root was identified by the appearance of aortic valve leaflets, and serial cross-sections of the entire aortic root area (5 μm thick with intervals of 50 μm) were mounted on 3-aminopropyl triethoxysilane-coated slides and stained with haematoxylin-phloxine-saffron (HPS). For each mouse, the lesion area was measured in 4 subsequent sections. Each section consisted of 3 segments (separated by the valves). For determination of atherosclerotic lesion severity, the lesions were classified into five categories according to the American Heart Association (AHA) criteria[24]: type 1 (early fatty streak), type 2 (regular fatty streak), type 3 (mild plaque), type 4 (moderate plaque), and type 5 (severe plaque). The total lesion area was calculated per cross-section. Due to a technical error one mouse of the OSM (30 μg/kg) was excluded from analysis. Lesion severity was calculated as relative amount of type I-V lesions in which the lesion-free segments are included. From this, the relative amounts of lesion-free segments and diseased segments were calculated, and the relative amount of diseased segments was further subdivided into type I–V lesions, where types I-III refer to mild, and types IV-V to severe lesions. Lesion composition of type IV and V lesions was assessed after double immunostaining with anti-α smooth muscle actin (1:400; PROGEN Biotechnik GmbH, Germany) for smooth muscle cells (SMC), and anti-mouse MAC-3 (1:400; BD Pharmingen, the Netherlands) for macrophages. Anti-α smooth muscle actin was labeled with Vina green (Biocare Medical, Pacheco, USA), and MAC-3 with DAB (Vector laboratories, Burlingame, USA). After slides were scanned and analyzed, cover slips were detached overnight in xylene and Sirius Red staining for collagen was performed. The necrotic area was measured in the Sirius Red-stained slides. Lesion stability index, as the ratio of collagen and αSMC area (i.e. stabilization factors) to macrophage and necrotic area (i.e. destabilization factors) was calculated as described previously[22]. Lesion composition was assessed in all type IV-V lesions with a mean of 5.9 ± 3.1 lesions in control, 5.6 ± 2.5 lesions in OSM 10 μg/kg/d, 2.9 ± 2.0 lesions in OSM 30 μg/kg/d temporary and 2.8 ± 2.9 lesions in OSM 30 μg/kg/d. Eight mice were excluded from analysis as there were no type IV-V lesions present (n = 1 in control; n = 4 in OSM 30 μg/kg/d temporary and n = 3 in OSM 30 μg/kg/d). In each segment used for lesion quantification, ICAM-1 expression and the number of monocytes adhering to the endothelium were counted after immunostaining with mouse monoclonal ICAM-1 antibody (1:400; Santa Cruz Biotechnology, Dallas, USA) and AIA 31240 antibody (1:500; Accurate Chemical and Scientific, New York, USA) respectively[25]. NLRP3 expression in the macrophages was quantified after staining with rabbit polyclonal antibody to NLRP3 (1:400; Abcam, Cambridge, UK). All slides were scanned by an Aperio AT2 slide scanner (Leica Biosystems). Atherosclerotic area, monocyte adherence and ICAM-1 expression were measured in Image Scope (version 12-12-2015), and the area that stained positive for αSMA, MAC-3, Sirius Red and NLRP3 in the plaques was quantified automatically in Fiji (version 30-5-2017) using a threshold method.

### Flow cytometry

To analyze the different monocyte subsets, 25 μL whole blood was incubated with antibodies against CD11b (APC-eFluor780-conjugated, eBioscience, San Diego, California, USA), Ly-6C (eFluor450-conjugated, eBioscience, San Diego, California, USA) and Ly-6G (A647-conjugated, Biolegend, San Diego, California, USA) for 30 min at RT. Erythrocytes were lysed with lysis buffer (deionized water with 168 mM ammonium chloride (Merck, Darmstadt, Germany), 9.99 mM potassium bicarbonate (Merck, Darmstadt, Germany) and 0.11 mM Na2EDTA (Sigma-Aldrich, St. Louis, MO, USA)) for 10 min on ice and remaining erythrocytes were lysed with fresh lysis buffer for 5 min on ice. After washing, cells were fixed in 1% paraformaldehyde for 10 min on ice, measured with flow cytometry (Gallios, Beckman Coulter Fullerton, CA, USA) and analyzed with Kaluza Flow Analysis Software Version 2.1 (Beckman Coulter). Monocytes were defined as CD11b^+^Ly-6G^-^.

### Statistics

BiKE transcriptomic dataset analyses were performed with GraphPad Prism 6 and Bioconductor software using a linear regression model adjusted for age and gender and a two-sided Student’s t-test assuming non-equal deviation, with correction for multiple comparisons according to Bonferroni, as previously described[16]. Data is presented as mean ± SD and adjusted p<0.05 was considered to indicate statistical significance.

Prior to protein data analyses, we applied a Box-Cox transformation on the proteins to improve normality, symmetry and to maintain all protein variables on a similar scale[21]. For protein to protein correlation we used linear regression analysis. Given consistency in terms of sample handling including time from blood draw to processing, same personnel handling all specimens and the ethnic homogeneity of the Icelandic population we adjusted only for age and sex in all our regression analyses.

Mouse data analyses were performed with GraphPad Prism 7.04 and IBM SPSS v25.0. Data are presented as mean ± SD. Normally (Gaussian) distributed mouse parameters were analyzed with a t-test or one-way ANOVA and not normally distributed mouse parameters with a Kruskal-Wallis test followed by a Mann-Whitney U test if significant. A significant difference between the 16 week 10 and 16 week 30 μg/kg/day groups was considered as a dose-dependent difference. The rejection criteria were adjusted using a Bonferroni-Holm correction. Correlation between plaque size and Ly-6C^High^ monocytes was tested with a Pearson correlation. A two-tailed p-value of 0.05 was regarded statistically significant in all analyses.

Cox proportional hazards regression was used for post incident CHD and Kaplan-Meier plots were applied to display survival data.

## Results

### mRNAs coding for *OSM*, *OSMR* and *LIFR* are present in human atherosclerotic plaques

**Fig 1:**
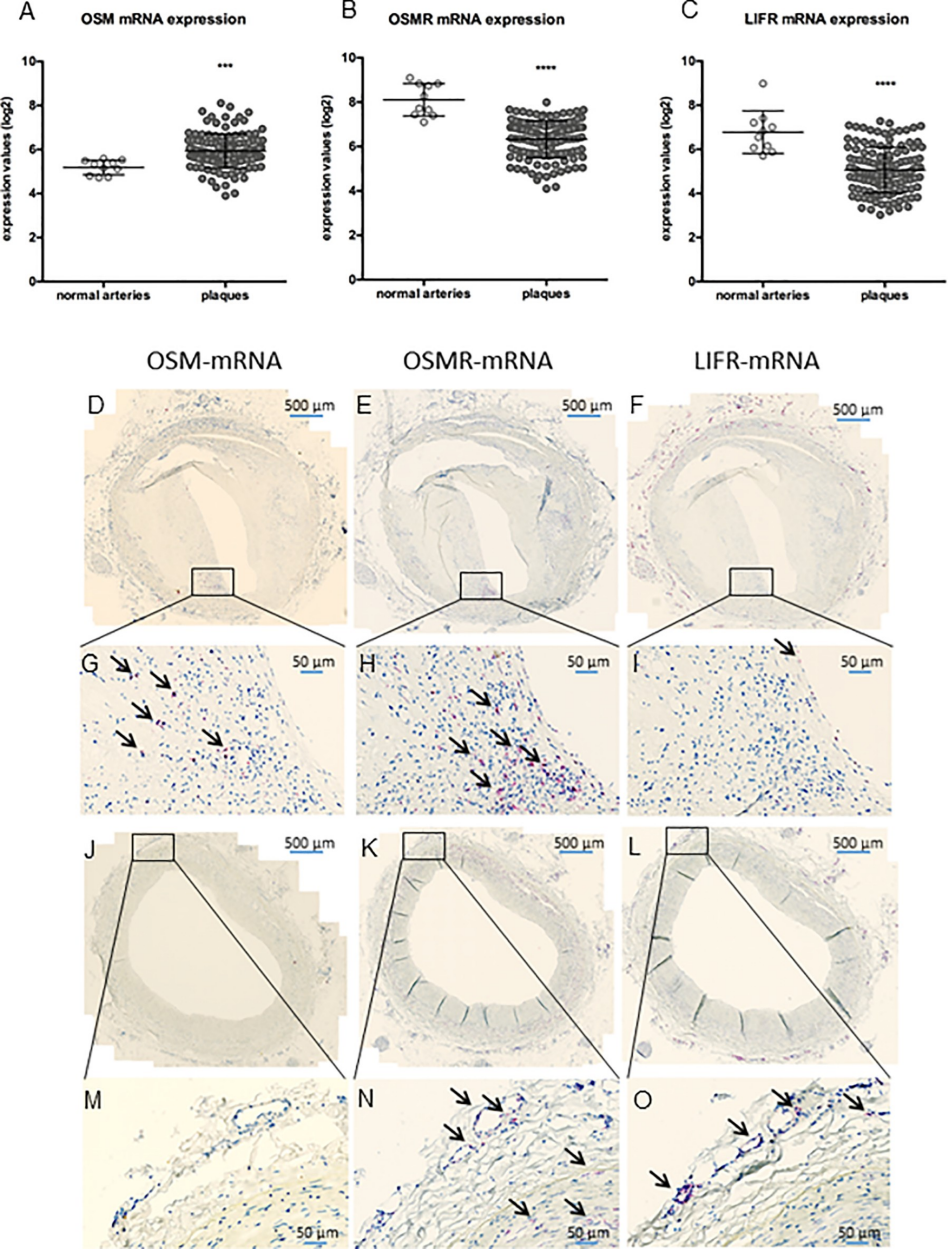
*OSM*, *OSMR* and *LIFR* mRNA expression is present in human atherosclerotic plaques. mRNA expression was measured in normal arteries and in carotid plaques by microarray analysis (A-C) and ISH was used to visualize *OSM*, *OSMR* and *LIFR* mRNA expression (red spots and shown by the black arrows) in two different stages of atherosclerosis development, the late fibroatheroma (D-I) and intimal xanthoma (J-O). A two-sided Student’s t-test assuming non-equal deviation was used to test for significance between normal and atherosclerotic arteries. ***p<0.001, ****p<0.0001.

To explore if OSM signaling can be involved in human plaque development, we first investigated if *OSM* mRNA and the mRNAs for the receptors for OSM, *OSMR* and *LIFR*, were present in late-stage human carotid plaques from the BiKE study. Gene expression analysis revealed presence of *OSMR*, *LIFR* and *OSM* mRNAs at low to moderate levels. mRNA expression of both receptors was significantly downregulated in plaques (p<0.0001) compared to normal arteries, while *OSM* expression was significantly increased (p = 0.003) (Fig 1A–1C). *OSM* mRNA expression positively correlated with macrophage markers and negatively with SMC markers (S1 Table). Subsequent *in situ* hybridization confirmed the presence of *OSMR*, *LIFR* and *OSM* mRNAs in all investigated atherosclerotic plaque stages (Fig 1D–1O), which is reflected in S2 Table.

### OSM is associated with endothelial activation markers IL-6 and VCAM-1 in humans

**Fig 2:**
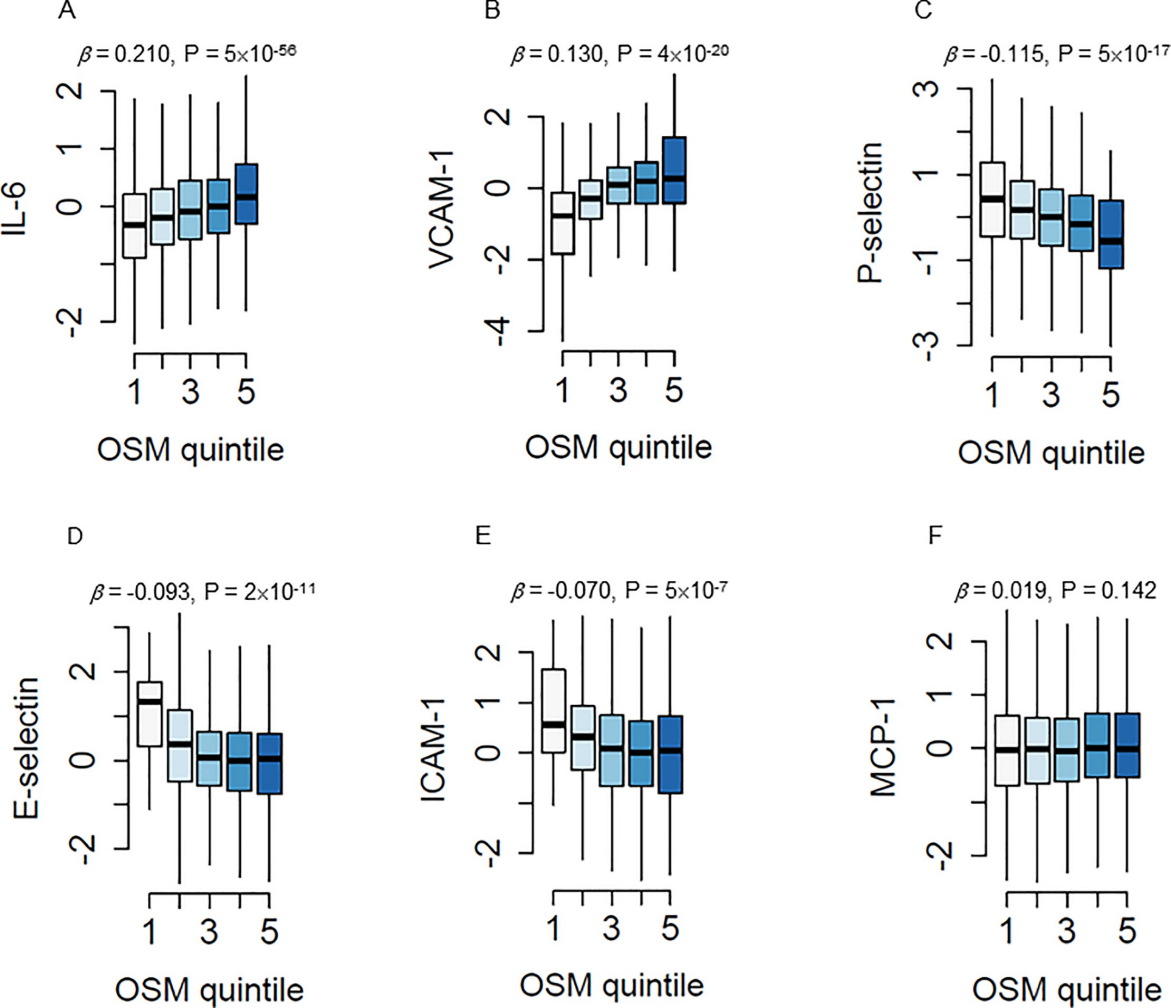
OSM is associated with endothelial activation markers. Association of serum IL-6 (A), VCAM-1 (B), P-selectin (C), E-selectin (D), ICAM-1 (E) and MCP-1 (F) levels (y-axis) with quintiles of increasing OSM serum levels (x-axis) using specific aptamers measured in 5457 subjects of the AGES cohort. Linear regression analyses were used to test for association.

We previously found that OSM induces endothelial activation both *in vitro* in human endothelial cells and *in vivo* in APOE*3Leiden.CETP mice[10]. To investigate if OSM can be linked with markers of endothelial activation in a human setting as well, we measured serum levels of OSM and several circulating endothelial activation markers in the AGES-Reykjavik study. OSM levels modestly correlated with IL-6 (β = 0.210, p = 5×10^−56^) and VCAM-1 (β = 0.130, p = 4×10^−20^) levels, but inversely with P-Selectin (β = -0.115, p = 5×10^−17^), E-Selectin (β = -0.092, p = 2×10^−11^) and ICAM-1 (β = -0.013, p = 5×10^−7^) levels (Fig 2). No correlation of OSM with MCP-1 was observed.

### Chronic exposure to OSM results in a pro-inflammatory vascular phenotype in APOE*3Leiden.CETP mice

**Fig 3:**
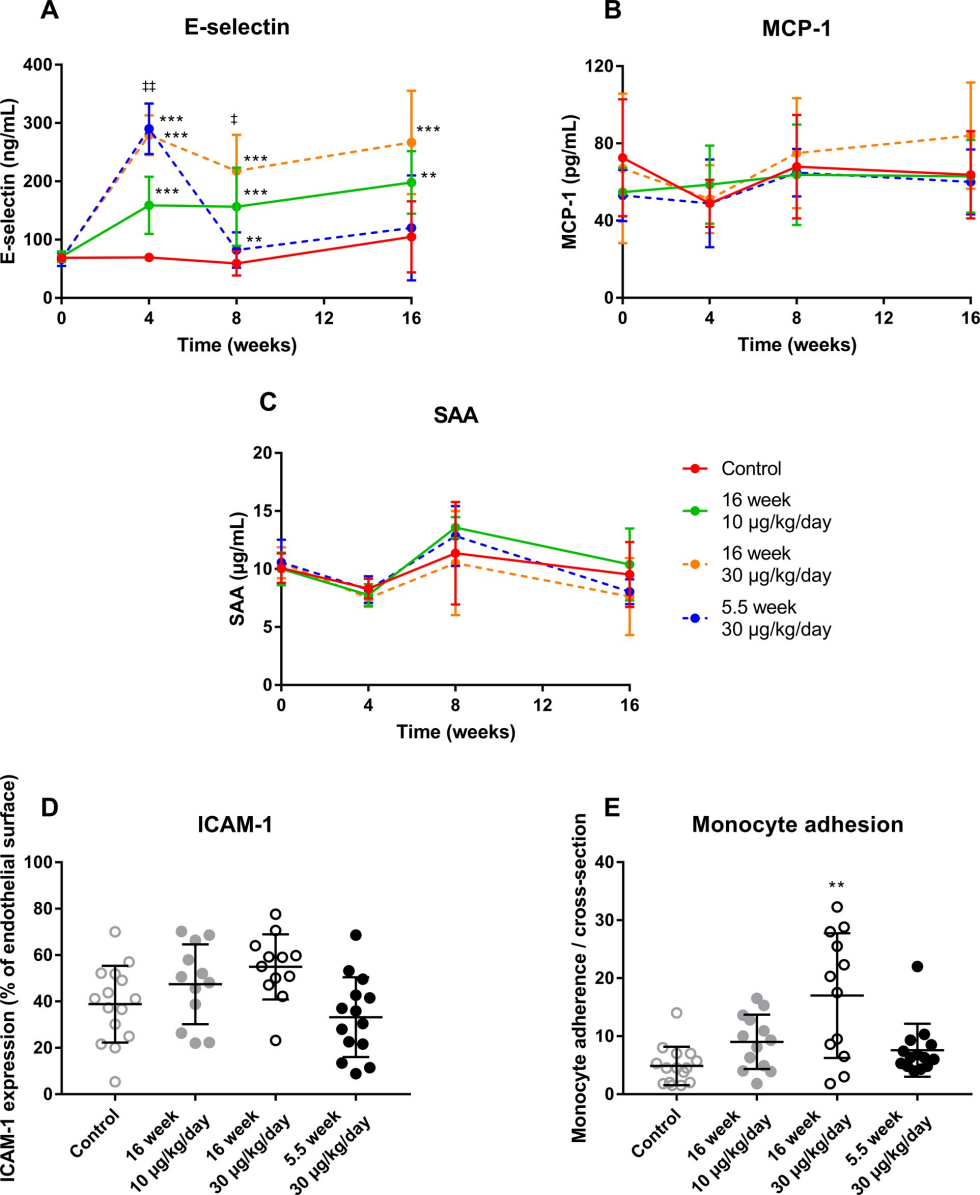
OSM induces a pro-inflammatory vascular phenotype in APOE*3Leiden.CETP mice. Plasma E-selectin, MCP-1 and SAA (A-C) were measured at multiple time points during the study. Monocyte adhesion (D) and endothelial ICAM-1 expression were assessed per cross-section in the aortic root area (E). Data represent mean ± SD (n = 12–20). The Kruskal-Wallis test was used to test for overall significance. If significant, the Mann-Whitney U test was performed to test which treatment groups were significantly different from the control group. Except for monocyte adhesion, which was tested with a t-test. Rejection criteria were adjusted using a Bonferroni-Holm correction. ‡ p<0.05 compared to 10 μg/kg/day; **p<0.01 compared to control; ‡‡ p<0.01 compared to 10 μg/kg/day; ***p<0.001 compared to control.

The above and our previous data[10] suggest a role for OSM in atherosclerosis development. Therefore, we performed a long-term study in which we administered OSM to APOE*3Leiden.CETP mice for 16 weeks. At t = 0, no difference in body weight was observed between the groups, but at t = 16, APOE*3Leiden.CETP mice treated with 30 μg/kg/day OSM for 16 weeks had a higher body weight than mice in the control group (p = 0.007) and mice treated with 10 μg/kg/day OSM for 16 weeks (p = 0.007). No difference in food intake was observed between the different groups. To specifically investigate the effect of OSM on the initiation of atherosclerosis, we added an initial priming group that was treated with OSM only for the first 5.5 weeks of the study. As previous studies had a much shorter duration (ranging from 6 hours to 3 weeks), we first investigated if long-term OSM treatment persistently causes an inflammatory phenotype by measuring E-selectin, MCP-1 and Serum amyloid A (SAA) plasma levels, as markers of vessel wall, general and liver-derived inflammation. Treatment groups receiving either 10 μg/kg/day (p≤0.002) or 30 μg/kg/day (p<0.001) OSM for 16 weeks showed markedly increased E-selectin levels at all time points and a dose-dependent increase at t = 4 (p<0.01) and 8 weeks (p<0.01). The group receiving 5.5 weeks 30 μg/kg/day OSM treatment also showed markedly increased E-selectin levels at t = 4 (p<0.001), though after discontinuation of OSM treatment, E-selectin levels dropped and declined to similar levels as the control group. MCP-1 and SAA levels did not differ between the OSM treated groups and control (Fig 3A–3C). Also, no statistical difference was observed in ICAM-1 expression at the endothelium in the aortic root area (Fig 3D). In contrast, monocyte adhesion, as functional marker of endothelial activation, in the aortic root area was increased from 4.9 ± 3.3 monocytes per cross-section in the control group to 17.9 ± 10.7 in the 16 weeks 30 μg/kg/day group (p = 0.003) (Fig 3E). These results indicate that continuous OSM exposure results in a sustained pro-inflammatory vascular phenotype, even after 16 weeks of treatment.

### OSM reduces atherosclerotic lesion area and severity in APOE*3Leiden.CETP mice

**Fig 4:**
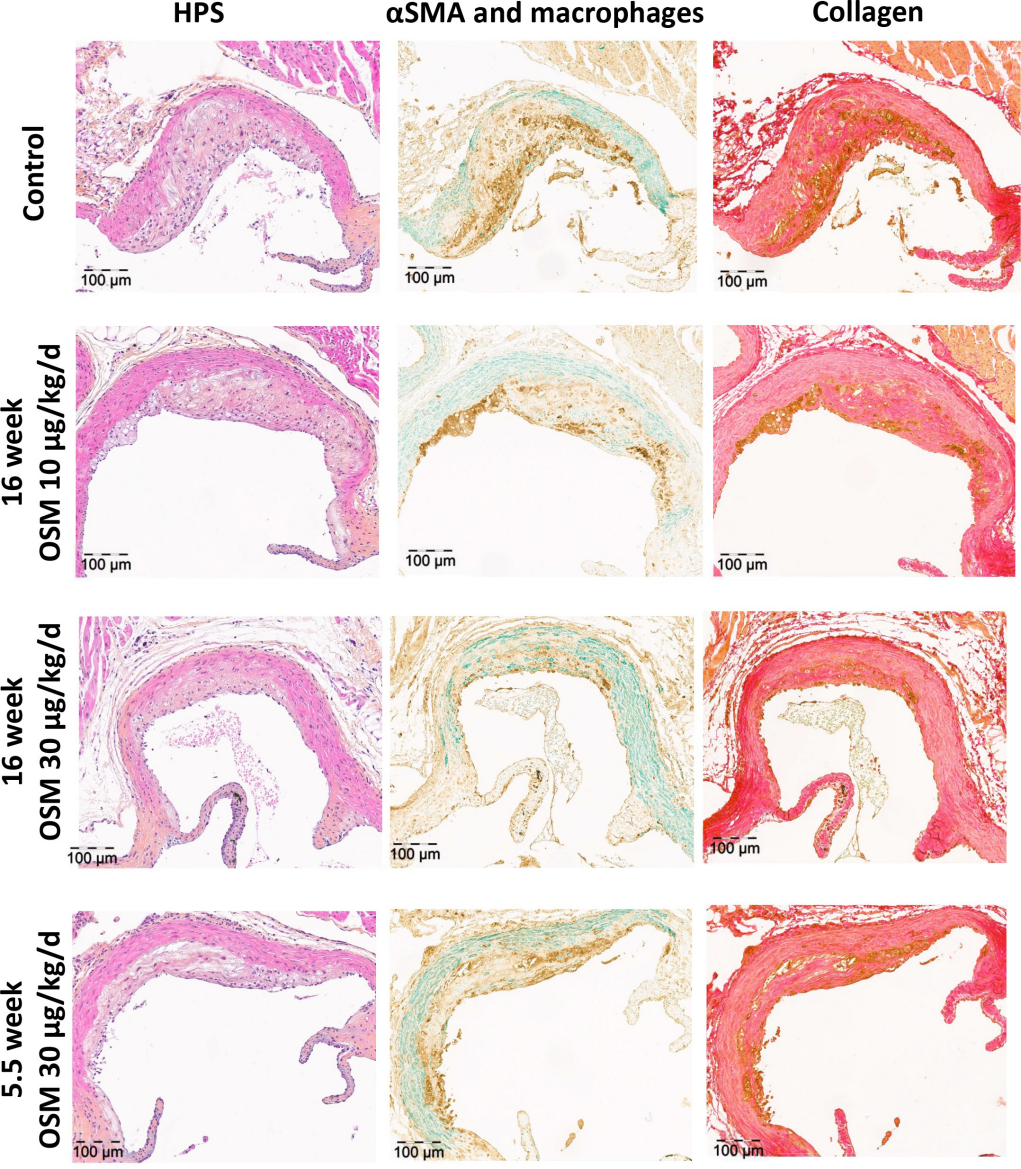
Effect of OSM on plaque composition in APOE*3Leiden.CETP mice. Representative pictures showing severe lesion types (type IV and V) stained with HPS staining, SMC staining (green), macrophage staining (brown) and collagen staining (red) to determine the effect of OSM on the lesion composition.

**Fig 5:**
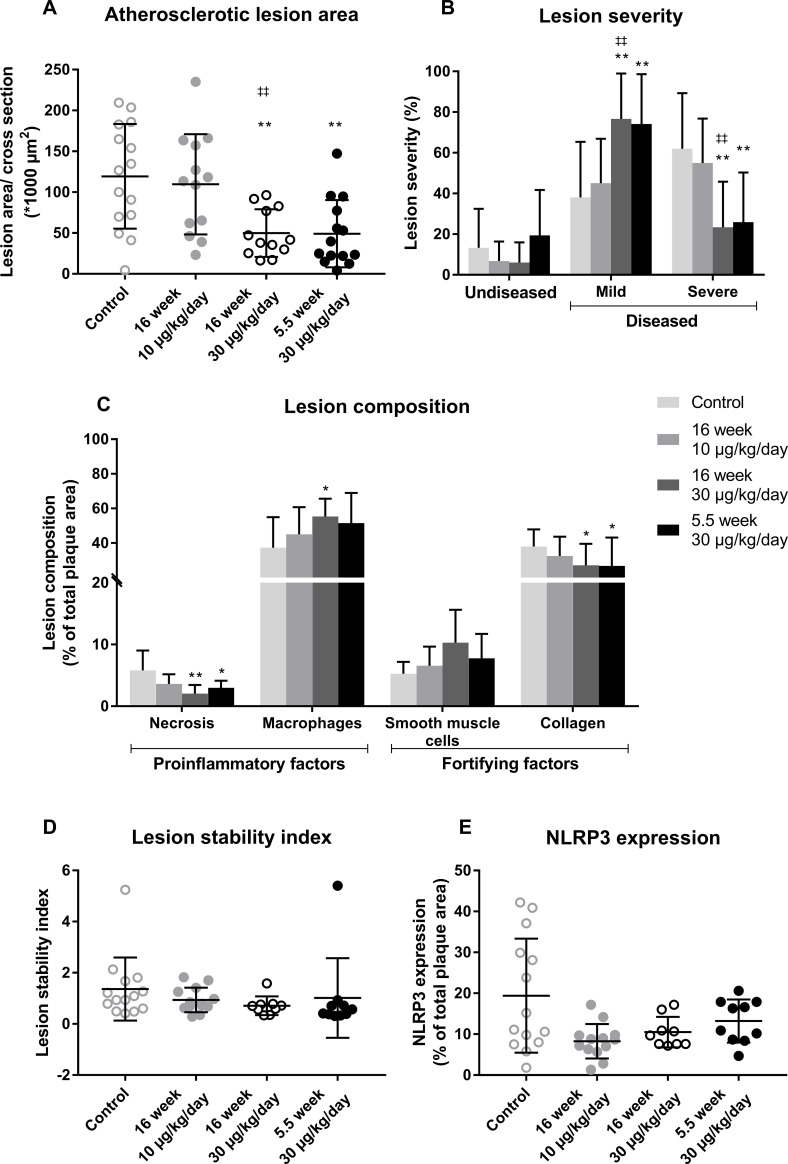
OSM reduces lesion size and severity in APOE*3Leiden.CETP mice. The atherosclerotic lesion size was determined in the aortic root area (A) and the lesions were classified as mild (type I-III) or severe (IV and V) lesions (B). Furthermore, the amount of necrosis, macrophages, smooth muscle cells and collagen was quantified (C) and the lesion stability index was calculated by dividing the summed proportions of SMCs and collagen, as stabilizing factors, by the summed proportions of necrosis and macrophages, as destabilizing factors (D). Additionally, the amount of NLRP3 expression was examined as percentage of the macrophage area (E). Data represent mean ± SD (n = 9–15). The t-test was used for statistical testing of the lesion area. The Kruskal-Wallis test was used to test all other parameters for overall significance. If significant, the Mann-Whitney U test was performed to test which treatment groups were significantly different from the control group. The rejection criteria were adjusted using a Bonferroni-Holm correction *p<0.05 **p<0.01 compared to control; ‡‡ p<0.01 compared to 10 μg/kg/day.

Total plasma cholesterol levels, a risk factor for cardiovascular disease, did not differ between any of the groups (S1 Fig). Atherosclerotic lesion size and severity were investigated in the aortic root area of which representative pictures are shown in Fig 4. The control group had an average lesion size of 119 ± 64 *1000 μm^2^. In the 5.5 week 30 μg/kg/day OSM group, plaque size was reduced by 59% (p = 0.002) and in the 16 week 30 μg/kg/day OSM group by 58% (p = 0.002), while the 16 week 10 μg/kg/day OSM treated group did not differ from the control (Fig 5A). The decrease in plaque area was dose-dependent (p = 0.006). In the control group, 62 ± 27% of the lesions were classified as severe lesions, while only 23 ± 22% (p = 0.001) and 26 ± 24% (p = 0.002) of the lesions were severe in the 16 week 30 μg/kg/day and 5.5 week 30 μg/kg/day OSM treated group, respectively. Again, the 16 week 10 μg/kg/day OSM treatment group did not differ from the control group. In line with plaque area, we observed a dose-dependent decrease in lesion severity (p = 0.003) (Fig 5B). Collectively, these results show that early continuous exposure to OSM reduces atherosclerotic lesion size and severity independently from plasma cholesterol in APOE*3Leiden.CETP mice.

### OSM has no effect on the stability of severe lesions in APOE*3Leiden.CETP mice

To assess the effect of OSM treatment on plaque stability of the severe lesions, we determined the amount of necrosis and macrophages, as indicators of unstable plaques and the amount of SMCs and collagen, as indicators of stable plaques (Fig 5C) in the severe lesions. Lesions in the control group consisted of 6 ± 3% necrosis, 37 ± 18% macrophages, 5 ± 2% SMCs and 38 ± 10% collagen. The amount of necrosis was decreased to 3 ± 1% in the 5.5 week 30 μg/kg/day OSM group (p = 0.012) and to 2 ± 1% in the 16 week 30 μg/kg/day OSM group (p = 0.01), while the macrophage content was slightly increased in the 16 week 30 μg/kg/day OSM group (55 ± 10%) (p = 0.016) only. The collagen content was decreased in the 5.5 week 30 μg/kg/day OSM group to 28 ± 17% (p = 0.012) and to 27 ± 13% in the 16 week 30 μg/kg/day OSM group (p = 0.018). No difference was observed in SMC content. The plaque composition of the 16 week 10 μg/kg/day OSM group was similar as in the control group. No differences were observed in the plaque stability ratio between the control and OSM treated groups (Fig 5D). As the amount of macrophages is not necessarily a measure for macrophage activity, we measured the expression of the caspase-1-activating inflammasome protein NLRP3 as marker of macrophage activation[26]. No significant difference was observed in NLRP3 expression in the plaque area (Fig 5E). In conclusion, although OSM does affect lesion composition by slightly increasing the amount of macrophages and decreasing the amount of necrosis and collagen, it does not affect overall plaque stability of the severe lesions.

### OSM reduces the inflammatory Ly-6C^High^ monocyte subset

**Fig 6:**
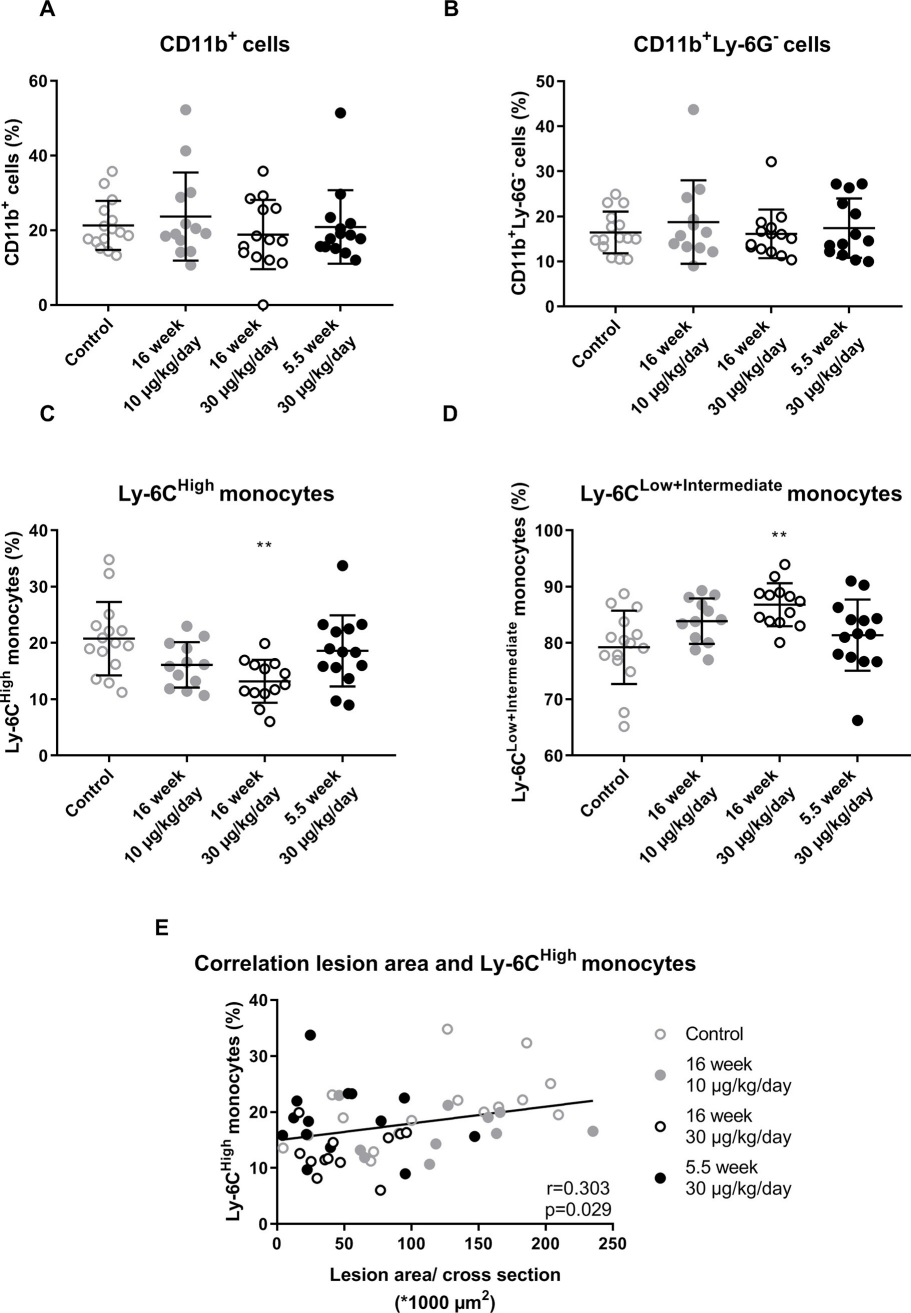
OSM reduces the percentage of circulating Ly-6C^High^ monocytes. No difference in percentage of CD11b^+^ cells (A) or CD11b^+^Ly-6G^-^ cells (B) was observed between the groups. But, ApoE*3Leiden.CETP mice treated with OSM have a higher percentage of circulating Ly-6C^High^ monocytes (C) and a lower percentage of circulating Ly-6C^Low+Intermediate^ monocytes (D). The percentage of Ly-6C^High^ monocytes was correlated with an increased lesion size (E). Data represent mean ± SD (n = 12–20). One-way ANOVA with Dunnett’s correction was used to test for significant differences between treatment groups and control. Pearson correlation was used to test the correlation between lesion size and Ly-6C^High^ monocytes. **p<0.01 compared to control.

No difference in the percentage of circulating CD11b^+^ cells or CD11b^+^Ly-6G^-^ cells was observed between the groups (Fig 6A and 6B). As the Ly-6C^High^ monocyte subset is linked to atherosclerosis development[27], we investigated the effect of OSM on the circulating monocyte subtype composition (S2 Fig). In the control group 20.8 ± 6.5% of the monocytes belonged to the Ly-6C^High^ subset and 79.2 ± 6.5% to the Ly-6C^Low+Intermediate^ subset. The amount of Ly-6C^High^ monocytes was decreased to 13.2 ± 3.8% in the 16 week 30 μg/kg/day OSM group (p = 0.004) and the amount of Ly-6C^Low+Intermediate^ monocytes increased to 86.8 ± 3.8% (p = 0.004) (Fig 6C and 6D). The Ly-6C^High^ subset showed a positive correlation with lesion size (r = 0.303, p = 0.029), supporting a role of the Ly-6C^High^ monocytes in the development of atherosclerosis (Fig 6E). Thus, OSM decreases the percentage of Ly-6C^High^ monocytes which may contribute to the smaller atherosclerotic lesion size.

### Serum OSM levels are associated with increased post incident CHD in humans

**Fig 7:**
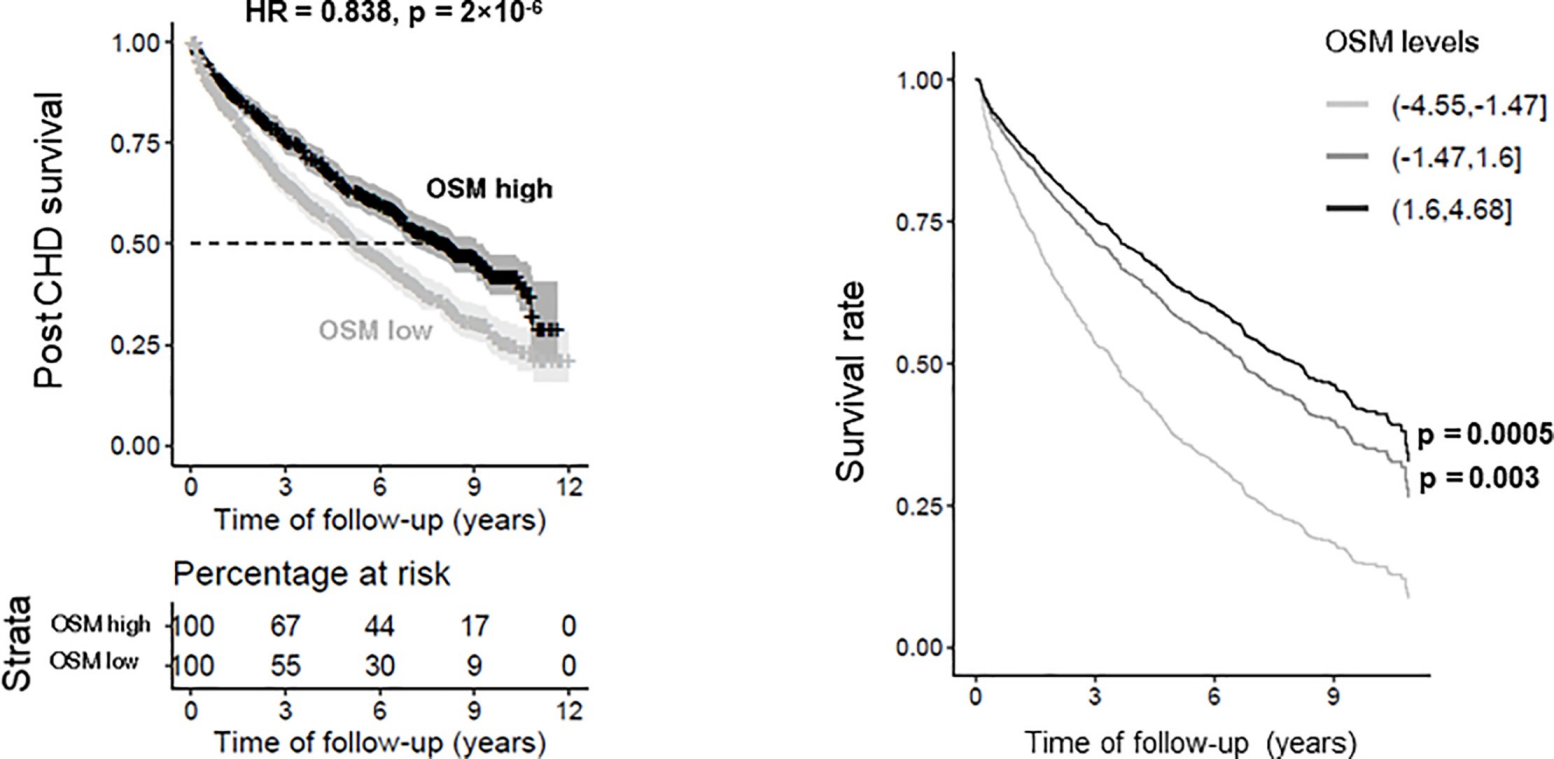
High OSM is associated with reduced post CHD mortality. Serum OSM levels of CHD patients were significantly associated with CHD related mortality rates when comparing the lower 25% quantile to the upper 75% quantile in OSM levels (hazard ratio (HR) = 0.838, p = 2×10^−6^) (A), and in the adjusted survival curves for the Cox model for three groups of OSM protein levels (top vs. bottom HR = 0.618, p = 0.0005) (B). Cox proportional hazards regression was performed to test for statistical significance and Kaplan-Meier plots were applied to display the survival data.

We next explored if variable levels of OSM in the human circulation were associated with survival probability in the AGES‐Reykjavik study. We found that higher serum OSM levels were associated with increased survival probability post incident CHD (HR = 0.838, p = 2×10^−6^) (Fig 7A), also using adjusted survival curves for the Cox model[28] (Fig 7B). Thus, elevated levels of OSM predicted reduced mortality in humans.

## Discussion

In the present study, we showed that mRNAs coding for *OSM* as well as its receptors, *OSMR* and *LIFR*, were expressed in human normal arteries and carotid atherosclerotic plaques. We demonstrated that serum OSM levels in humans were positively correlated with several but not with other well-known markers of endothelial activation. Chronic OSM administration to APOE*3Leiden.CETP mice reduced atherosclerotic lesion size and severity even after initial priming. In line with these data, increased OSM levels in humans were associated with decreased post incident CHD mortality.

Extending the previous finding by Albasanz-Puig *et al*[13], who showed that OSM is present in both human and murine atherosclerotic plaques, we here demonstrated the presence of *OSMR* and *LIFR* mRNA in human normal and atherosclerotic arteries as well. The relatively higher *OSMR* and *LIFR* expression in normal arteries compared to atherosclerotic arteries may be explained by the high expression of the receptors on endothelial and vascular SMCs[8,29]. These cells make up a relatively large proportion of the normal artery, but less of the atherosclerotic plaque, in which there is influx and proliferation of inflammatory cells, which might dilute *OSMR* and *LIFR* expression. The opposite can be reasoned for the increased *OSM* expression in atherosclerotic arteries, as OSM is mainly produced by activated macrophages and neutrophils[5,6,30]. Moreover, *OSMR* and *LIFR* expression may be downregulated in endothelial and SMCs in plaques compared to endothelial and SMCs in normal arteries. Besides, the chronic inflammatory state during atherosclerosis development drives vascular SMC differentiation, which reduces the expression of SMC specific markers[31] and may therefore also reduce expression of *LIFR* and *OSMR*. This contention is in line with our observation that OSM is negatively correlated with SMC markers and with Kakutani *et al*., who showed that OSM induces SMC differentiation[4].

The correlation of OSM with IL-6 and VCAM-1 in the AGES‐Reykjavik study is in line with previous findings *in vitro*[10]. However, the inverse association of OSM with E-selectin and ICAM-1 contradicts with previous data showing increased levels induced by OSM in human endothelial cells *in vitro*[10] and increased serum E-selectin levels in APOE*3Leiden.CETP mice. The absence of a positive correlation between OSM and ICAM-1, E-selectin and P-selectin may be caused by statin use in the AGES‐Reykjavik study (approx. 22%)[21], as statins reduce ICAM-1, E-selectin and P-selectin plasma levels in patients with coronary artery disease[32]. Regardless, mice treated with OSM in the present study did show increased serum E-selectin levels which dropped after discontinuation of OSM treatment, indicating a causal relationship between OSM and E-selectin *in vivo* in mice.

As our present study had a much longer duration than previous intervention studies with OSM in mice[9,10], we first verified if the previously observed short-term inflammatory state[10] is also present after 16 weeks of OSM administration. OSM increased plasma E-selectin levels and monocyte adhesion in the aortic root area, similarly as in our previous study[10], indicating that OSM induces a sustained inflammatory state even after long-term OSM treatment. Although inflammation has been reported to contribute to atherosclerosis development[33], our results show, to our knowledge for the first time, that long-term chronic OSM treatment independently of cholesterol-lowering, results in significantly smaller and less severe atherosclerotic lesions in APOE*3Leiden.CETP mice, clearly indicating that prolonged exposure to OSM has anti-atherogenic effects. Previously, Zhang *et al*., using a different approach, showed that OSMR deficient ApoE^-/-^ mice have smaller and more stable plaques than their OSMR expressing littermates[14], suggesting that signaling via the LIFR alone or prevention of IL-31 and OSM signaling through OSMR[34] has a similar beneficial effect.

No difference was observed in the lesion stability index, and although we observed a slight increase in the amount of macrophages as percentage of the total plaque area, the amount of NLRP3 expression was very low and did not differ between any of the groups, indicating that the pro-inflammatory macrophage activity was not affected[26]. In line with this, the percentage of pro-inflammatory Ly-6C^High^ monocytes[35] was decreased and the percentage of non-inflammatory Ly-6C^Low+Intermediate^ monocytes, which actively patrol the luminal site of the endothelium where they remove debris and damaged cells and are associated with reparative processes[35], was increased in OSM treated mice. The decrease in Ly-6C^High^ monocytes plausibly contributes to the attenuated atherosclerosis development.

Although our findings are counter-intuitive with several previously described pro-inflammatory characteristics of OSM[9,36], they are in line with studies addressing the anti-inflammatory properties of OSM. It has been shown that OSM administration suppresses TNFα[37] and IL-1β release *in vitro*[38], whereas TNFα, IL-1β and IFN-γ expression is increased in adipose tissue of OSMR knockout mice[39]. Both cytokines are involved in atherosclerosis progression in mice as TNFα promotes atherosclerosis[40] and IL-1β knockout mice have smaller and less severe atherosclerotic lesions[41]. In humans, anti-inflammatory treatments targeting TNFα or IL-1β are associated with decreased risk of myocardial infarction and overall cardiovascular events[42,43]. Collectively, these and our data indicate that OSM has anti-inflammatory effects as well which may contribute to its anti-atherogenic properties. Moreover, OSM has been reported to induce endothelial proliferation[12,44] and to increase expression of adhesion molecules that bind endothelial progenitor cells[45,46], suggesting that OSM stimulates replacement of leaky, dysfunctional endothelial cells by new and healthy endothelial cells[47] and may therefore attenuate atherogenesis in the initial stages of the disease. This contention is in line with our finding that mice treated with OSM for only 5.5 weeks had a similar lesion size and severity as mice receiving OSM during a 16 week period and suggests that the observed anti-atherogenic effects of OSM have taken place during the initial stages of atherosclerosis development. Furthermore, although the observed increase in SMCs observed in this study was not significant, others have reported that OSM significantly enhances SMC proliferation *in vitro*[13], which is a contributor to a stable plaque phenotype[48]. Finally, the anti-atherogenic effects of OSM that were observed in this study might be caused by OSM-induced alterations in estrus cycling. In human breast cancer cell lines, OSM was shown to suppress oestrogen receptor-α expression[49], which may affect atherosclerosis development as estrogens increase VLDL production[50], and might thereby contribute to an atherogenic phenotype[51]. However, no changes in total cholesterol and increased triglyceride levels were observed in OSM treated mice (S1 Fig), making this possibility unlikely. Since OSM affects many different cell types and the mice in this study received OSM systemically, it is important to note that the observed effects on atherosclerosis development could also be caused by indirect effects of OSM. To conclude, OSM may contribute to attenuation of plaque development and improvement of plaque severity by: (1) its anti-inflammatory properties, (2) regenerating the endothelial barrier, (3) induction of SMC proliferation, (4) reducing the pro-inflammatory monocyte phenotype and promoting a more regenerative phenotype[48] and (5) indirectly by asserting its effects on other cell types that are involved in atherosclerosis development.

The anti-atherogenic effect of OSM in APOE*3Leiden.CETP mice is consistent with the increased post incident CHD survival probability in humans with higher OSM levels in the AGES‐Reykjavik study. Similarly, OSM treatment increased survival in a mouse injury model of acute myocardial infarction[52], emphasizing the regenerative properties of this cytokine[44,53].

As OSM has been suggested to have a progressive effect in chronic inflammatory diseases such as, RA[54] and inflammatory bowel disease[36,55], it has been proposed as a possible pharmaceutical target to suppress inflammation in these diseases[36,54,55] and the effect of anti-OSM treatment in RA has already been investigated in a phase 2 clinical trial[54]. However, considering the anti-atherogenic effects and positive effect of OSM on survival in the present study, we strongly recommend that cardiovascular disease markers and survival are carefully monitored when testing an OSM inhibiting approach. In addition, since this study shows that OSM has beneficial immune modulating effects, the role of OSM in inflammatory diseases possibly needs to be reconsidered.

Taken together, our study provides more insight into the role of OSM in atherosclerosis development. APOE*3Leiden.CETP mice treated with OSM had smaller and less severe plaques associated with a decrease in pro-inflammatory Ly-6C^High^ monocytes. In line with the favorable effect in mice, we found an increased survival probability in humans that have high OSM levels, suggesting an atheroprotective effect for OSM.

## Supporting information

S1 Table Correlation between OSM and genes of interest in plaques. Pearson correlation analyses were calculated from n = 127 human plaque microarrays, p-values are corrected for multiple comparisons according to the Bonferroni method. *p<0.05, **p<0.01, ***p<0.001, ****p<0.0001. Correlation considered weak if r < 0.3 moderate if 0.3 < r < 0.5 and strong if r > 0.5.
(DOCX)

S2 Table Quantification of ISH signal in various atherosclerotic plaque stages. The amount of ISH signal was scored in various atherosclerotic plaque stages. A general score and a single cell score was given. 0 = No signal, 1 = Few cells expressing mRNA, 2 = Low expression, 3 = Moderate expression and 4 = High expression.
(DOCX)

S3 Table Effect of OSM on body weight and food intake. At t = 0, no difference in body weight was observed between the groups. At t = 16, APOE*3Leiden.CETP mice treated with 30 μg/kg/day OSM for 16 weeks had a higher body weight than mice in the control group and mice treated with 10 μg/kg/day OSM for 16 weeks. No difference in food intake was observed between the different groups. Body weight at t = 0 was normally distributed and therefore analyzed with a One-way ANOVA, while body weight at t = 16 was not normally distributed and therefore analyzed with the Kruskal-Wallis test with subsequent Mann-Whitney U tests to test which groups were significantly different from the control group and to test if there was a dose-dependent effect. Food intake was measured with the Kruskal-Wallis test as too little data points were available to evaluate the distribution of the data. The rejection criteria were adjusted using a Bonferroni-Holm correction. **p<0.01 compared to control; ‡‡ p<0.01 compared to 10 μg/kg/day.
(DOCX)

S1 Fig OSM does not affect total plasma cholesterol levels and increases triglyceride levels in APOE*3Leiden.CETP mice. Total plasma cholesterol (A) and triglyceride (B) levels were measured at multiple time points during the study. Data represent mean ± SD (n = 13–20). The Kruskal-Wallis test was used to test for overall significance. If significant, the Mann-Whitney U test was performed to test which treatment groups were significantly different from the control group.
(DOCX)

S2 Fig Representative pictures of the distribution of the Ly-6C monocyte subsets. Based on the Ly-6C expression, monocytes were distributed into 3 monocyte subsets, the Ly-6C^Low^, Ly-6C^Intermediate^ and Ly-6C^High^ monocyte subset.
(DOCX)
