# Supplementary material for: Oncostatin M reduces atherosclerosis development in APOE*3Leiden.CETP mice and is associated with increased survival probability in humans
Source: PLoS One. 2019 Aug 28;14(8):e0221477. doi: 10.1371/journal.pone.0221477 (PMC6713386; doi:10.1371/journal.pone.0221477)
Supplement: S1 Table — Pearson correlation analyses were calculated from n = 127 human plaque microarrays, p-values are corrected for multiple comparisons according to the Bonferroni method. *p<0.05, **p<0.01, ***p<0.001, ****p<0.0001. Correlation considered weak if r < 0.3 moderate if 0.3 < r < 0.5 and strong if r > 0.5. (DOCX) [file pone.0221477.s001.docx]

**S1 Table. Correlation between OSM and genes of interest in plaques.**

|  | **Gene symbol** | **Pearson r** | **p-value** | **Significance level** |
| --- | --- | --- | --- | --- |
| **Cell type markers** | | | | |
| *Smooth muscle cells* | | | | |
| Myosin heavy chain 11 | MYH11 | -0.4327 | < 0.0001 | **** |
| Smoothelin | SMTN | -0.4437 | < 0.0001 | **** |
| Alpha smooth muscle actin | ACTA2 | -0.3476 | < 0.0001 | **** |
| Myocardin | MYOCD | -0.4119 | < 0.0001 | **** |
| Transgelin | TAGLN | -0.3127 | 0.0004 | *** |
| *Endothelial cells* | | | | |
| von Willebrand factor | VWF | 0.1486 | 0.0967 | ns |
| Pecam-1 (CD31) | PECAM1 | 0.3009 | 0.0006 | *** |
| *Dendritic cells* | | | | |
| Itgax (CD11c) | ITGAX | 0.4738 | < 0.0001 | **** |
| Ly75 (CD205) | LY75 | -0.03098 | 0.7295 | ns |
| CD80 | CD80 | 0.6013 | < 0.0001 | **** |
| *T Lymphocytes* | | | | |
| CD11b | ITGAM | 0.4048 | < 0.0001 | **** |
| ITGAL | ITGAL | 0.5012 | < 0.0001 | **** |
| CD27 | CD27 | 0.107 | 0.233 | ns |
| CD28 | CD28 | 0.2859 | 0.0012 | ** |
| CD3 delta | CD3D | 0.3678 | < 0.0001 | **** |
| CD4 | CD4 | 0.1078 | 0.2295 | ns |
| CD8A | CD8A | 0.2258 | 0.0107 | * |
| PTPRC (CD45RA) | PTPRC | 0.3758 | < 0.0001 | **** |
| CD69 | CD69 | 0.4909 | < 0.0001 | **** |
| ITGAE | ITGAE | 0.2827 | 0.0013 | ** |
| FABP4 | FABP4 | 0.3884 | < 0.0001 | **** |
| *Macrophages* | | | | |
| CD83 | CD83 | 0.5474 | < 0.0001 | **** |
| CD86 | CD86 | 0.4934 | < 0.0001 | **** |
| CD163 | CD163 | 0.4434 | < 0.0001 | **** |
| TNFRSF9 | TNFRSF9 | 0.3696 | < 0.0001 | **** |
| CD40 | CD40 | 0.3422 | < 0.0001 | **** |
| CD36 | CD36 | 0.4466 | < 0.0001 | **** |
| **Inflammation/ Apoptosis Calcification markers** | | | | |
| IL-1beta | IL1B | 0.5657 | < 0.0001 | **** |
| NFkB | NFKB1 | 0.1764 | 0.0481 | * |
| MCP-1 | CCL2 | 0.5311 | < 0.0001 | **** |
| Caspase-3 | CASP3 | 0.2726 | 0.002 | ** |
| Caspase-7 | CASP7 | 0.05738 | 0.5233 | ns |
| Caspase-9 | CASP9 | 0.2318 | 0.009 | ** |
| BCL2 | BCL2 | 0.2761 | 0.0018 | ** |
| RANTES | CCL5 | 0.3821 | < 0.0001 | **** |
| BMP4 | BMP4 | -0.1434 | 0.1091 | ns |
| **Extracellular matrix/ degradation** | | | | |
| MMP9 | MMP9 | 0.4202 | < 0.0001 | **** |
| TIMP1 | TIMP1 | 0.3891 | < 0.0001 | **** |
| **Growth factors** | | | | |
| TGFB1 | TGFB1 | 0.4113 | < 0.0001 | **** |
| TGFA | TGFA | 0.328 | 0.0002 | *** |
| IGF1 | IGF1 | 0.256 | 0.0038 | ** |
| PDGFA | PDGFA | -0.02346 | 0.7943 | ns |
| PDGFB | PDGFB | 0.2417 | 0.0064 | ** |
| PDGFC | PDGFC | -0.2382 | 0.0072 | ** |
| PDGFD | PDGFD | -0.2889 | 0.001 | ** |
| **Chemokines and receptors** | | | | |
| Interferon gamma | IFNG | 0.2032 | 0.0225 | * |
| IL2 | IL2 | 0.2446 | 0.0058 | ** |
| IL4 | IL4 | 0.03414 | 0.7043 | ns |
| IL5 | IL5 | 0.1947 | 0.0289 | * |
| IL6 | IL6 | 0.5659 | < 0.0001 | **** |
| IL9 | IL9 | 0.05453 | 0.5442 | ns |
| IL10 | IL10 | 0.4213 | < 0.0001 | **** |

Pearson correlation analyses were calculated from n=127 human plaque microarrays, p-values are corrected for multiple comparisons according to the Bonferroni method. *p<0.05, **p<0.01, ***p<0.001, ****p<0.0001. Correlation considered weak if r < 0.3 moderate if 0.3 < r < 0.5 and strong if r > 0.5.
