# Supplementary material for: Oncostatin M reduces atherosclerosis development in APOE*3Leiden.CETP mice and is associated with increased survival probability in humans
Source: PLoS One. 2019 Aug 28;14(8):e0221477. doi: 10.1371/journal.pone.0221477 (PMC6713386; doi:10.1371/journal.pone.0221477)
Supplement: S2 Table — The amount of ISH signal was scored in various atherosclerotic plaque stages. A general score and a single cell score was given. 0 = No signal, 1 = Few cells expressing mRNA, 2 = Low expression, 3 = Moderate expression and 4 = High expression. (DOCX) [file pone.0221477.s002.docx]

|  |  | **mRNA expression** | | |
| --- | --- | --- | --- | --- |
|  |  | **OSM** | **OSMR** | **LIFR** |
| **Adaptive Intimal Thickening** | **Neo-intima** | 1 | 2 | 2 |
|  | **Media** | 1 | 3 | 2 |
|  | **Adventitia** | 1 | 3 | 3 |
| **Intimal Xanthoma** | **Neo-intima** | 1 | 2 | 2 |
|  | **Media** | 0 | 2 | 3 |
|  | **Adventitia** | 0 | 2 | 3 |
| **Pathological Intimal Thickening** | **Neo-intima** | 1 | 2 | 2 |
|  | **Media** | 1 | 3 | 2 |
|  | **Adventitia** | 1 | 3 | 3 |
| **Early Fibroatheroma** | **Neo-intima** | 2 | 2 | 2 |
|  | **Media** | 1 | 2 | 2 |
|  | **Adventitia** | 1 | 3 | 3 |
| **Late Fibroatheroma** | **Neo-intima** | 2 | 2 | 2 |
|  | **Media** | 2 | 2 | 2 |
|  | **Adventitia** | 1 | 2 | 4 |
| **Fibrous Calcified Plaque** | **Neo-intima** | 2 | 2 | 2 |
|  | **Media** | 1 | 3 | 2 |
|  | **Adventitia** | 1 | 3 | 2 |

**S2 Table. Quantification of ISH signal in various atherosclerotic plaque stages.**

The amount of ISH signal was scored in various atherosclerotic plaque stages. A general score and a single cell score was given. 0 = No signal, 1 = Few cells expressing mRNA, 2 = Low expression, 3 = Moderate expression and 4 = High expression.
