# Supplementary material for: Oncostatin M reduces atherosclerosis development in APOE*3Leiden.CETP mice and is associated with increased survival probability in humans
Source: PLoS One. 2019 Aug 28;14(8):e0221477. doi: 10.1371/journal.pone.0221477 (PMC6713386; doi:10.1371/journal.pone.0221477)
Supplement: S3 Table — At t = 0, no difference in body weight was observed between the groups. At t = 16, APOE*3Leiden.CETP mice treated with 30 μg/kg/day OSM for 16 weeks had a higher body weight than mice in the control group and mice treated with 10 μg/kg/day OSM for 16 weeks. No difference in food intake was observed between the different groups. Body weight at t = 0 was normally distributed and therefore analyzed with a One-way ANOVA, while body weight at t = 16 was not normally distributed and therefore analyzed with the Kruskal-Wallis test with subsequent Mann-Whitney U tests to test which groups were significantly different from the control group and to test if there was a dose-dependent effect. Food intake was measured with the Kruskal-Wallis test as too little data points were available to evaluate the distribution of the data. The rejection criteria were adjusted using a Bonferroni-Holm correction. **p<0.01 compared to control; ‡‡ p<0.01 compared to 10 μg/kg/day. (DOCX) [file pone.0221477.s003.docx]

**S3 Table. Effect of OSM on bodyweight and food intake.**

| **Treatment group** | **Bodyweight t=0** | **Bodyweight t=16** | **Food intake** |
| --- | --- | --- | --- |
|  | (g) | (g) | (g/mouse/day) |
| Control | 21.1 ± 1.2 | 23.5 ± 1.9 | 3.0 ± 0.1 |
| 16 week 10 µg/kg/day | 21.1 ± 1.8 | 23.3 ± 1.5 | 3.0 ± 0.1 |
| 16 week 30 µg/kg/day | 21.1 ± 1.5 | 24.8 ± 1.2** ‡‡ | 3.2 ± 0.1 |
| 5.5 week 10 µg/kg/day | 21.1 ± 1.5 | 22.9 ± 1.4 | 3.1 ± 0.1 |
| p-value | 0.995 | 0.007 | 0.169 |

At t=0, no difference in bodyweight was observed between the groups. At t=16, ApoE*3Leiden.CETP mice treated with 30 µg/kg/day OSM for 16 weeks had a higher bodyweight than mice in the control group and mice treated with 10 µg/kg/day OSM for 16 weeks. No difference in food intake was observed between the different groups. Bodyweight at t=0 was normally distributed and therefore analyzed with a One-way ANOVA, while bodyweight at t=16 was not normally distributed and therefore analyzed with the Kruskal-Wallis test with subsequent Mann-Whitney U tests to test which groups were significantly different from the control group and to test if there was a dose-dependent effect. Food intake was measured with the Kruskal-Wallis test as too little data points were available to evaluate the distribution of the data. The rejection criteria were adjusted using a Bonferroni-Holm correction. **p<0.01 compared to control; ‡‡ p<0.01 compared to 10 µg/kg/day.
