# Supplementary material for: Oncostatin M reduces atherosclerosis development in APOE*3Leiden.CETP mice and is associated with increased survival probability in humans
Source: PLoS One. 2019 Aug 28;14(8):e0221477. doi: 10.1371/journal.pone.0221477 (PMC6713386; doi:10.1371/journal.pone.0221477)
Supplement: S1 Fig — Total plasma cholesterol (A) and triglyceride (B) levels were measured at multiple time points during the study. Data represent mean ± SD (n = 13–20). The Kruskal-Wallis test was used to test for overall significance. If significant, the Mann-Whitney U test was performed to test which treatment groups were significantly different from the control group. (DOCX) [file pone.0221477.s004.docx]

**S1 Fig. OSM does not affect total plasma cholesterol levels and increases triglyceride levels in APOE*3Leiden.CETP mice.** Total plasma cholesterol (A) and triglyceride (B) levels were measured at multiple time points during the study. Data represent mean ± SD (n=13-20). The Kruskal-Wallis test was used to test for overall significance. If significant, the Mann-Whitney U test was performed to test which treatment groups were significantly different from the control group.
