# Supplementary material for: Oncostatin M reduces atherosclerosis development in APOE*3Leiden.CETP mice and is associated with increased survival probability in humans
Source: PLoS One. 2019 Aug 28;14(8):e0221477. doi: 10.1371/journal.pone.0221477 (PMC6713386; doi:10.1371/journal.pone.0221477)
Supplement: S2 Fig — Based on the Ly-6C expression, monocytes were distributed into 3 monocyte subsets, the Ly-6CLow, Ly-6CIntermediate and Ly-6CHigh monocyte subset. (DOCX) [file pone.0221477.s005.docx]

**
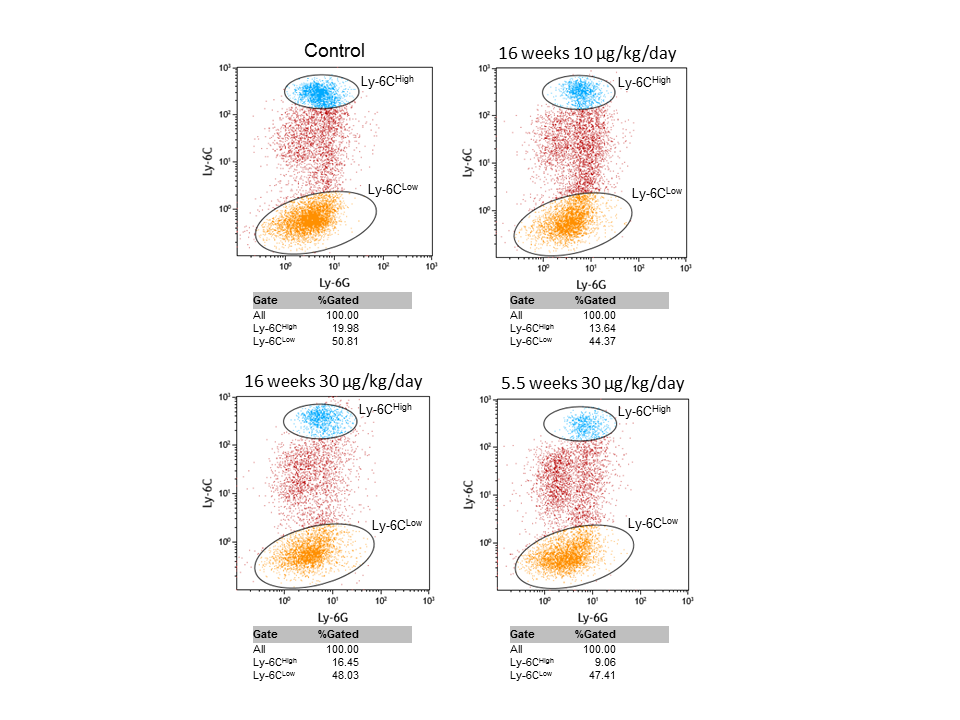
**

**S2 Fig. Representative pictures of the distribution of the Ly-6C monocyte subsets.** Based on the Ly-6C expression, monocytes were distributed into 3 monocyte subsets, the Ly-6C^Low^, Ly-6C^Intermediate^ and Ly-6C^High^ monocyte subset.
